# Hypoxemia Index Associated with Prehospital Intubation in COVID-19 Patients

**DOI:** 10.3390/jcm9093025

**Published:** 2020-09-20

**Authors:** Romain Jouffroy, Romain Kedzierewicz, Clement Derkenne, Kilian Bertho, Marine Scannavino, Benoit Frattini, Frederic Lemoine, Daniel Jost, Bertrand Prunet

**Affiliations:** Paris Fire Brigade, Emergency Medicine Department, 1 Place Jules Renard, 75017 Paris, France; romain.kedzierewicz@pompiersparis.fr (R.K.); clement.derkenne@pompiersparis.fr (C.D.); kilian.bertho@pompiersparis.fr (K.B.); marine.scannavino@pompiersparis.fr (M.S.); benoit.frattini@pompiersparis.fr (B.F.); frederic.lemoine@pompiersparis.fr (F.L.); daniel.jost@pompiersparis.fr (D.J.); bertrand.prunet@pompiersparis.fr (B.P.)

**Keywords:** COVID-19, prehospital, intubation, hypoxemia, index, association

## Abstract

Background: There exists a need for prognostic tools for the early identification of COVID-19 patients requiring prehospital intubation. Here we investigated the association between a prehospital Hypoxemia Index (HI) and the need for intubation among COVID-19 patients in the prehospital setting. Methods: We retrospectively analyzed COVID-19 patients initially cared for by a Paris Fire Brigade advanced life support (ALS) team in the prehospital setting between 8th March and 18th April of 2020. We assessed the association between HI and prehospital intubation using receiver operating characteristic (ROC) curve analysis and logistic regression model analysis after propensity score matching. Results are expressed as odds ratio (OR) and 95% confidence interval (CI). Results: We analyzed 300 consecutive COVID-19 patients (166 males (55%); mean age, 64 ± 18 years). Among these patients, 45 (15%) were deceased on the scene, 34 (11%) had an active care restriction, and 18 (6%) were intubated in the prehospital setting. The mean HI value was 3.4 ± 1.9. HI was significantly associated with prehospital intubation (OR, 0.24; 95% CI: 0.12–0.41, *p* < 10^−3^) with a corresponding area under curve (AUC) of 0.91 (95% CI: 0.85–0.98). HI significantly differed between patients with and without prehospital intubation (1.0 ± 1.0 vs. 3.6 ± 1.8, respectively; *p* < 10^−3^). ROC curve analysis defined the optimal HI threshold as 1.3. Bivariate analysis revealed that HI <1.3 was significantly, positively associated with prehospital intubation (OR, 38.38; 95% CI: 11.57–146.54; *p* < 10^−3^). Multivariate logistic regression analysis demonstrated that prehospital intubation was significantly associated with HI (adjusted odds ratio (ORa), 0.20; 95% CI: 0.06–0.45; *p* < 10^−3^) and HI <3 (ORa, 51.08; 95% CI: 7.83–645.06; *p* < 10^−3^). After adjustment for confounders, the ORa between HI <1.3 and prehospital intubation was 3.6 (95% CI: 1.95–5.08; *p* < 10^−3^). Conclusion: An HI of <1.3 was associated with a 3-fold increase in prehospital intubation among COVID-19 patients. HI may be a useful tool to facilitate decision-making regarding prehospital intubation of COVID-19 patients initially cared for by a Paris Fire Brigade ALS team. Further prospective studies are needed to confirm these preliminary results.

## 1. Background

The first cases of COVID-19 were described in Asia in late 2019 [1,2,3,4], and on 11 March 2020, the World Health Organization declared this disease to be a worldwide pandemic [5]. Although the overall mortality rate is low [6,7,8,9], to date, COVID-19 has caused ~200,000 deaths worldwide, half of which are in Europe. COVID-19 is caused by infection with SARS-CoV-2 [10], and ~25% of patients suffer a severe form of this disease [6].

The most severe form of COVID-19 involves acute respiratory failure (ARF) due to hypoxia and hypoxemia, which sometimes necessitates intubation and mechanical ventilation prior to hospital intensive care unit (ICU) admission. We previously reported that dyspnea is the main symptom requiring prehospital treatment by the Paris Fire Brigade prehospital emergency service [11]. One peculiarity of respiratory SARS-CoV-2 infection is the presentation of a low respiratory rate (RR) increase despite severe hypoxemia reflected by low pulse oximetry (SpO_2_) of variable depth depending on the stage of the disease, along with a lack of functional signs and respiratory distress signs [11]. This remarkable disconnect in rest between profound hypoxemia and proportional signs of respiratory distress was named “happy hypoxia” by Dhont et al. [12].

The COVID-19 pandemic poses a risk of an inadequate ratio between needs (patients with severe COVID-19 ARF requiring support ventilation) and resources (available medical ventilator devices). Thus, it would be useful to develop a simple tool for early assessment of prehospital intubation requirement—for example, something similar to the shock index, which is calculated as the ratio between heart rate and systolic blood pressure [13] and serves as a simple clinical tool allowing early recognition of sepsis in the emergency department [14]. This tool could be useful to physicians for the decision-making process according to evidenced-based medicine knowledge without being polluted by an infodemic and the spread of fake news about COVID-19 [15].

In the present study, we report the relationship between COVID-19 patients intubated in the prehospital setting by a Paris Fire Brigade advanced life support team and the Hypoxemia Index (HI), defined as the ratio between initial pulse oximetry and initial respiratory rate.

## 2. Methods

### 2.1. Design, Setting, and Participants

As previously described [11], the prehospital Paris Fire Brigade emergency medical system is a two-tiered response system—comprising a basic life support (BLS) tier served by 200 teams of 3–5 professional rescuers deployed from 77 stations and an advanced life support (ALS) tier served by 44 ambulance teams, each including an emergency physician, a nurse, and a driver [16].

Emergency calls are assessed by a dispatch center operator, who may decide to send a BLS and/or ALS team on the basis of the clinical history and symptoms reported by the patient or witness. Once rescue teams have arrived on the scene, the emergency physician examines the patient, and then the patient can either be left on the scene, admitted to the emergency department (ED), or admitted directly to the intensive care unit (ICU), depending on the level of criticality.

Here we performed a retrospective observational study that included patients who required intervention by a Paris Fire Brigade team (BLS and/or ALS) between 8th March and 18th April of 2020. No exclusion criteria were applied.

### 2.2. Ethical Considerations

This retrospective study was approved by the French Society of Anaesthesia and Intensive Care ethics committee on 7 April 2020 (Ref: IRB 00010254-2020-055).

### 2.3. Data Collection

To minimize the bias in data abstraction [17], data collection was performed by a single investigator using a previously established standardized abstraction template. From ALS prehospital medical reports, we retrieved the patients’ demographic characteristics (age and gender), medical history (previous hypertension, cardiopathy, coronaropathy, chronic renal failure, chronic obstructive pulmonary disease, diabetes mellitus, obesity, stroke, immunosuppression, asthma, and active smoking), initial (i.e., at the first medical contact) prehospital vital sign values (systolic blood pressure, heart rate (HR), pulse oximetry (SpO_2_), respiratory rate (RR), temperature, and Glasgow coma scale (GCS)), and record of administered prehospital treatments (oxygen modality and catecholamine type and dose). We also recorded the date of suspected contamination, the date of first symptoms, and the date of contact. The COVID-19 diagnosis was established after transfer to the hospital, and prehospital diagnosis was based on a bundle of arguments including clinical signs and recent contact with a COVID-19 patient.

The Hypoxemia Index (HI) was calculated as the ratio between the initial SpO_2_ (%), i.e., SpO_2_ on room air, and the initial RR (breaths per minute): $HI=\frac{SpO2}{RR}$.

### 2.4. Statistical Analyses

Results are expressed as mean and standard deviation for quantitative parameters with a normal distribution, as median and interquartile range (Q1–Q3) for parameters with a non-Gaussian distribution, and as absolute value and percentage for qualitative parameters. All analyses were performed using R 3.4.2 (http://www.R-project.org; the R Foundation for Statistical Computing, Vienna, Austria).

First, we performed bivariate analyses evaluating the relationship between covariates and prehospital intubation. Second, we analyzed the prehospital HI level as a continuous variable and as a binary variable using the optimal threshold defined by receiver operating characteristic (ROC) curve analysis, i.e., the optimal threshold with the highest sensitivity and the highest specificity associated with prehospital intubation, using the Youden index [18]. To limit the impact of outliers, and to enable provision of more robust presentations, we obtained an adjusted average ROC curve by averaging 10,000 bootstrapped samples (sampling with replacement). We compared the HI and SpO_2_ curves using the De Long method [19].

Third, we assessed the relationship between HI and prehospital intubation using logistic regression—including the following potential confounders: age, hypertension, cardiopathy, coronaropathy, chronic renal failure, chronic obstructive pulmonary disease, diabetes mellitus, obesity, immunosuppression, asthma, active smoking, systolic blood pressure, and HR—based on previous studies and physiopathological knowledge [6,9,11,19,20]. The results are expressed as adjusted odds ratio (ORa) and 95% confidence interval (CI).

Fourth, to reduce the potential effect of confounders, we performed a propensity score analysis. We estimated the propensity score, i.e., the probability of HI lower than the optimal threshold, using logistic regression on the basis of the following potential confounders: age, hypertension, cardiopathy, coronaropathy, chronic renal failure, chronic obstructive pulmonary disease, diabetes mellitus, obesity, stroke, immunosuppression, asthma, active smoking, systolic blood pressure, and HR [21]. We used nearest-neighbor matching to match patients on the basis of the logit of the propensity score [22], and then we assessed the balance of covariates based on absolute mean differences. After matching, the baseline characteristics included in the propensity score were compared between cases (prehospital intubation) and controls (no prehospital intubation) using paired tests to reduce the influence of sample size on *p* value, with a threshold of 10% considered acceptable [23]. To estimate the average treatment effect, the ORa and 95% CI of prehospital intubation was evaluated for a value lower than the optimal threshold.

## 3. Results

### 3.1. Study Characteristics

**Figure 1:**
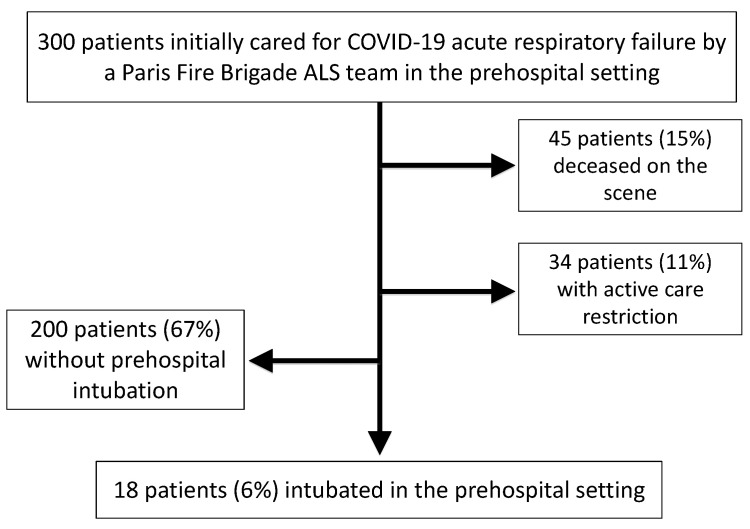
Patient flowchart.

Between 8th March and 18th April of 2020, a total of 300 consecutive patients suffering from COVID-19 were attended to by a prehospital Paris Fire Brigade ALS team. The mean age was 64 ± 18 years, and 165 patients (55%) were male. A total of 45 patients (15%) were deceased on the scene, 30 (66%) of whom were male. The deceased patients were significantly older than the alive patients (70 ± 14 years vs. 63 ± 19 years, respectively; *p* = 0.018). Among the patients alive on the scene, 34 (11% of all patients) had an active care restriction in the prehospital setting. These 34 patients included 20 males (59%) and were significantly older than the living patients without an active care restriction (81 ± 9 years vs. 62 ± 18 years, respectively; *p* < 10^−3^). Finally, 18 patients (6%) required prehospital intubation (Figure 1).

Table 1 summarizes the population demographics and clinical characteristics, and Table 2 summarizes the main prehospital functional symptoms.

### 3.2. Main Measurements

**Figure 2:**
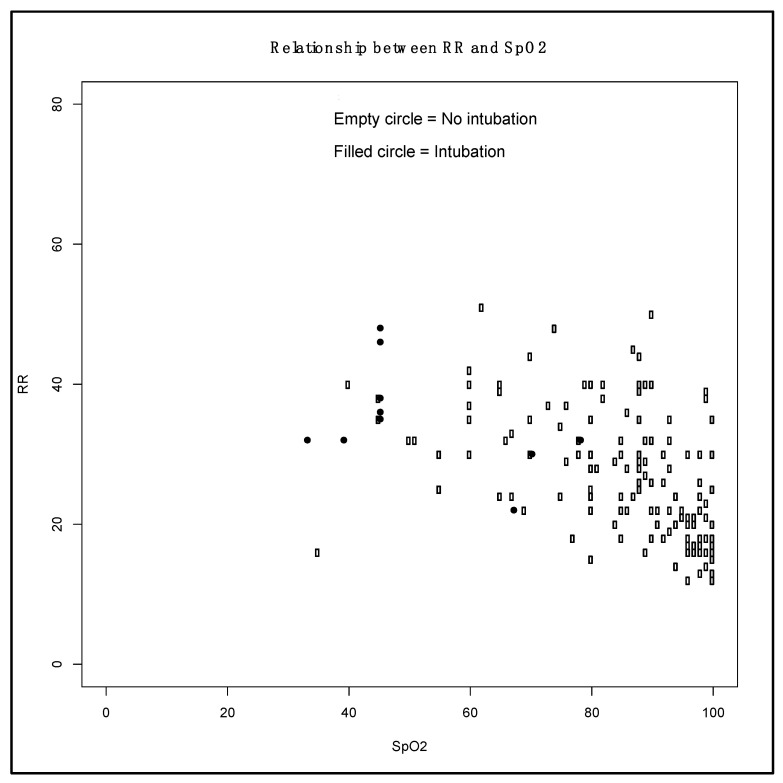
Relationship between initial respiratory rate (RR) and initial pulse oximetry (SpO_2_) in intubated and non-intubated patients.

In the overall population, the initial RR was 28 ± 10 bpm and initial SpO_2_ was 89% (95% CI: 76–98%). Patients with and without prehospital intubation showed significantly different initial values for RR (27 ± 10 bpm vs. 35 ± 11 bpm, respectively; *p* = 0.005) and SpO_2_ (90% (80–98%) vs. 45% (43–56%), respectively; *p* < 10^−3^). Figure 2 illustrates the relationship between initial RR and initial SpO_2_ in intubated and non-intubated patients.

A total of 215 patients (72%) did not require prehospital intubation and instead received a median oxygen flow supplementation of 15 L/min (95% CI: 9–15 L/min). All prehospital intubations were performed by physicians wearing an FFP2 facial mask, glasses, and non-sterile gloves.

**Figure 3:**
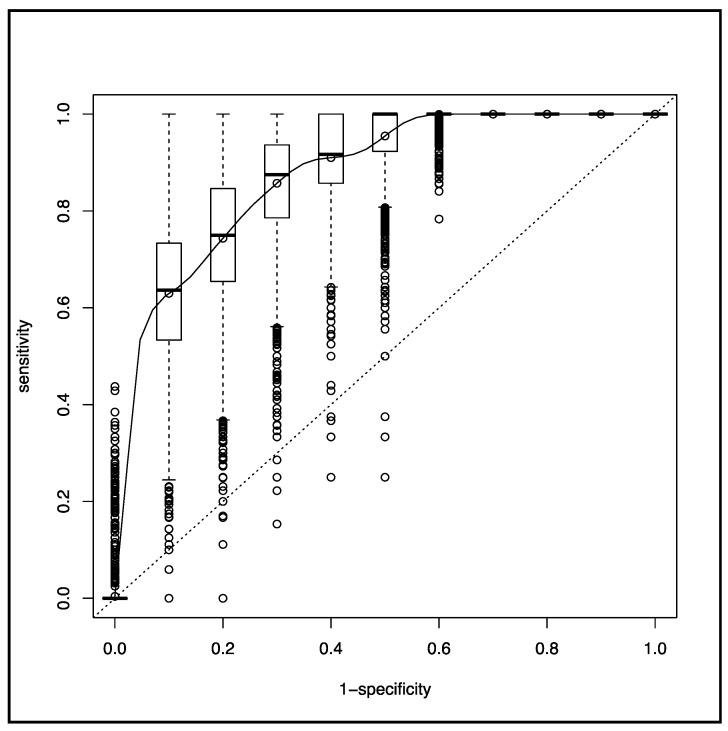
ROC curve of the pulse oximetry (SpO_2_).

Eighteen patients (6%) needed prehospital intubation after anesthesia induction based on ketamine (*n* = 14, 78%) or etomidate (*n* = 4, 12%) and succinylcholine (*n* = 18, 100%). Sedation was maintained by an association of midazolam and sufentanyl (*n* = 18, 100%). Fifteen patients (83%) were paralyzed with atracurium. After intubation, their median RR was 15 bpm (12–20 bpm), median inspired fraction of oxygen was 100% (80–100%), median end-expiratory pressure was 12 cm H_2_O (5–15 cm H_2_O), and mean tidal volume was 442 ± 71 mL, corresponding to an indexed ideal body weight of 6–8 mL.kg^−1^. HI was significantly associated with prehospital intubation (OR, 0.24; 95% CI: 0.12–0.41; *p* < 10^−3^), with a corresponding AUC of 0.91 (0.85–0.98). The SpO_2_ ROC curve is depicted in Figure 3, and the corresponding AUC was 0.87 (0.77–0.98) without a significant difference from the HI AUC (*p* = 0.41).

**Figure 4:**
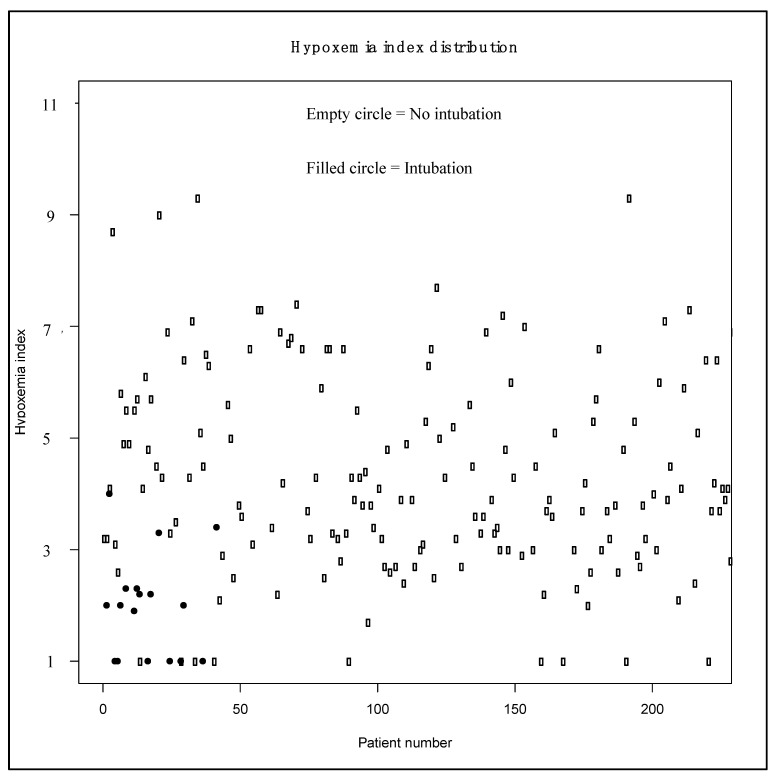
Hypoxemia Index (HI) distribution between intubated and non-intubated patients.

**Figure 5:**
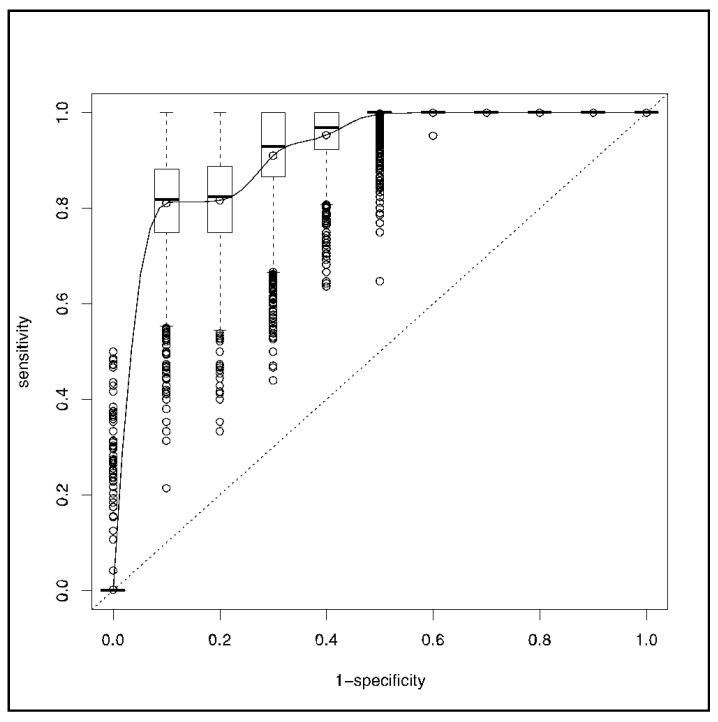
ROC (receiver operating characteristic) curve of the Hypoxemia Index (HI).

The HI significantly differed (*p* < 10^−3^) between patients with and without prehospital intubation: 1.0 ± 1.0 vs. 3.6 ± 1.8, respectively. Figure 4 illustrates the HI distribution among intubated and non-intubated patients, and Figure 5 shows the ROC curve for HI.

The HI optimal threshold, i.e., the threshold with the highest sensitivity and the highest specificity using the Youden index, was 1.3. In bivariate analysis, an HI of <1.3 was significantly, positively associated with prehospital intubation (OR, 38.38; 95% CI: 11.57–146.54; *p* < 10^−3^) (Table 1). Multivariate logistic regression revealed that prehospital intubation was significantly associated with HI (ORa, 0.20; 95% CI: 0.06–0.45; *p* < 10^−3^) and with HI <1.3 (ORa, 51.08; 95% CI: 7.83–645.06; *p* < 10^−3^).

### 3.3. Propensity Score Matching Analysis

**Figure 6:**
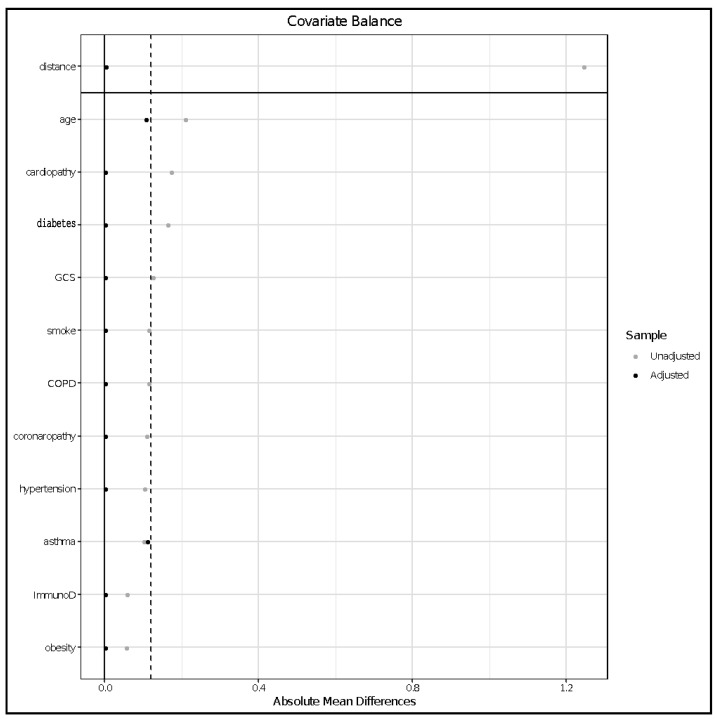
Standardized mean deviation between cases and controls after matching. COPD = chronic obstructive pulmonary disease, GCS = Glasgow coma scale, SBP = systolic blood pressure, ImmunoD = immunosuppression, smoke = active smoking.

Figure 6 shows the absolute mean differences between subgroups after matching.

After adjustment for confounders, HI of <1.3 was significantly associated with prehospital intubation (ORa, 3.6; 95% CI: 1.95–5.08; *p* < 10^−3^).

## 4. Discussion

In the present analysis of 300 COVID-19 patients attended to by a prehospital Paris Fire Brigade ALS team, we observed that HI was significantly associated with prehospital intubation. Specifically, an HI of <1.3 was associated with a 3-fold increase in prehospital intubation.

Patients who required prehospital intubation were those with lower initial SpO_2_ and higher initial RR values. The severe respiratory form of COVID-19 appears as acute respiratory failure (ARF) with a notable discrepancy between the increases of RR and SpO_2_. As previously reported [11], the respiratory form of COVID-19 differs from other types of ARF commonly encountered in the prehospital setting [24,25], with COVID-19 patients commonly exhibiting a relatively low SpO_2_ value but with no physical signs of acute respiratory distress (e.g., cyanosis and intercostal and substernal retractions).

ARF treatment is symptomatically treated because, despite many studies, no etiological treatments are yet available [26,27,28]. Symptomatic ARF treatment is based on oxygen therapy—ranging from low-flow or high-flow nasal cannula therapy [29] to invasive mechanical ventilation [30,31]. In ARF COVID-19 disease, the optimal ventilatory mode remains controversial [30,31,32] due to the lack of scientific evidence and concerns about healthcare provider contamination through SARS-CoV-2 aerosolization [33,34,35,36,37]. It is presently unclear how to choose the adequate ventilatory mode for each patient, with the goals being to avoid excessive intubation and to not delay necessary invasive mechanical ventilation. This is a great challenge to the prehospital management of ARF during the COVID-19 pandemic, due to the unfavorable ratio between needs and resources. Early identification of patients at high risk of unfavorable respiratory evolution in the prehospital setting would help avoid both under-triage and over-triage [38,39].

In COVID-19 ARF, clinical signs may not adequately indicate severity [11]; thus, there is a need to explore other means of severity assessment. Outside the hospital, hypoxemia depth may be assessed by arterial blood gas analysis, especially since COVID-19 patients exhibit lower partial arterial oxygen pressure(PaO2) than SpO_2_ [12]. However, during the COVID-19 pandemic, there may be a shortage of blood gas analysis medical devices for prehospital emergency services. Thus, severity assessment based on physical examination should also be available in order to counteract the previous limitations. This is why we have developed and tested the Hypoxemia Index to identify patients with a higher risk of requiring prehospital intubation.

The present study has several limitations that restrict the generalization of our conclusions. First, it was a retrospective study with a restricted number of patients intubated in the prehospital setting. Second, there is the possibility of bias from misclassification of covariates since data were collected from prehospital reports. Third, the study design and the statistical analyses do not permit any conclusion regarding the causal link between HI and prehospital intubation; the results only indicate an association. Fourth, as the relationship between RR and SpO_2_ was not linear [11], we cannot conclude that the relationship between HI and prehospital intubation is linear; consequently, it may affect the interpretation of HI, especially since HI is markedly influenced by SpO2. So far, HI should not be used, prior to a prospective validation, as a decisional trigger for prehospital intubation. Fifth, data were collected by a single investigator, potentially compromising data accuracy [40]. Sixth, no pediatric patients were included in our analyses, and thus our results may not be transposable to a pediatric population, particularly since SARS-CoV-2 does not affect children as it affects adults [41]. Seventh, SpO_2_ is not the best tool to reflect PaO2, which mostly triggered dyspnea. Eighth, this study was performed in a single city within one prehospital emergency system. Ninth, intubation of COVID-19 patients remains challenging and under debate [40], with risk for healthcare providers [42,43] and patients [44,45,46,47]. Consequently, the decision-making for intubation may be delayed until arrival at a high-level care facility with an experienced team member [48,49].

Beyond these limitations, our results appear to indicate that HI may be a useful tool for distinguishing COVID-19 patients with a higher risk of prehospital intubation in a prehospital setting.

## 5. Conclusions

The presently described Hypoxemia Index is associated with the requirement for prehospital intubation in COVID-19 patients cared for in the prehospital setting. This Hypoxemia Index may be useful for screening COVID-19 patients for risk associated with prehospital intubation, but these results must be confirmed by prospective studies.

## Figures and Tables

**Table 1:** Population demographics and clinical characteristics.

	Overall Population (*n* = 300)	In-Hospital Admission without Prehospital Intubation (*n* = 200)	Prehospital Intubation (*n* = 18)	OR (95% CI)	*p* Value
**Demographics**					
Age in years	64 ± 18	61 ± 19	62 ± 11	1.00 (0.98–1.03)	0.798
Male gender	166 (55%)	161 (80%)	5 (28%)	0.46 (0.14–0.57)	<10^−3^
Hypertension	91 (30%)	82 (41%)	9 (50%)	0.36 (0.58–4.41)	0.359
Coronaropathy	22 (7%)	22 (11%)	0 (0%)	0.1 (0.01–8.8 × 10^31^)	0.991
Cardiopathy	34 (11%)	33 (17%)	1 (6%)	0.31 (0.02–1.61)	0.267
Diabetes mellitus	56 (19%)	48 (24%)	8 (44%)	2.78 (0.99–7.66)	0.046
Obesity	36 (12%)	32 (16%)	4 (22%)	1.60 (0.43–4.85)	0.438
COPD	22 (7%)	22 (11%)	0 (0%)	0.1 (0.01–8.8 × 10^31^)	0.991
Chronic renal failure	2 (0.1%)	0 (0%)	2 (11%)	2.06 × 10^8^ (0.1–8.8 × 10^31^)	0.991
Immunosuppression	15 (5%)	13 (7%)	2 (11%)	1.90 (0.28–7.76)	0.467
Asthma	22 (7%)	20 (10%)	2 (11%)	1.19 (0.18–4.63)	0.828
Active smoking	21 (7%)	21 (11%)	0 (0%)	0.1 (0.01–7.4 × 10^32^)	0.991
**Prehospital vital signs**					
HR (beats/min)	96 ± 23	95 ± 23	108 ± 18	1.02 (0.99–1.04)	0.024
SBP (mm Hg)	138 ± 24	138 ± 24	143 ± 26	1.01 (0.99–1.03)	0.411
Body core temperature (°C)	37.1 (36.1–38.2)	37.0 (36.1–38.2)	38.0 (36.6–38.7)	1.3 (0.94–1.88)	0.156
Glasgow coma scale	15 (15–15)	15 (15–15)	14 (3–15)	0.76 (0.68–0.85)	
RR (movements/min)	28 ± 10	27 ± 10	35 ± 11	1.06 (1.02–1.11)	0.011
Pulse oximetry (%)	89 (76–98)	90 (80–98)	45 (43–56)	0.90 (0.85–0.94)	<10^−3^
HI	3.4 ± 1.9	3.6 ± 1.8	1.0 ± 1.0	0.24 (0.12–0.41)	<10^−3^
HI <1.3	20 (7%)	9 (5%)	11 (61%)	38.38 (11.57–146.54)	<10^−3^
Norepinephrine administration	7 (2%)	3 (0.1%)	4 (22%)	18.76 (3.80–103.48)	<10^−3^

SBP = systolic blood pressure, HR = heart rate, RR = respiratory rate, HI = Hypoxemia Index, COPD = chronic obstructive pulmonary disease, OR = odds ratio, 95% CI = 95 per cent confidence interval. Results are expressed as mean ± standard deviation for quantitative parameters with normal distribution, as median (interquartile range) for quantitative parameters with non-Gaussian distribution, and as absolute value (percentage) for qualitative parameters. *p* values correspond to the univariate odds ratio between prehospital intubation and no-prehospital intubation patients.

**Table 2:** Main prehospital functional symptoms collected from advanced life support (ALS) prehospital medical reports.

Symptom	*n*	Percentage (%)
Dyspnea	152	51
Fever	135	45
Cough	92	31
Chest pain	50	17
Myalgia	32	11
Discomfort	28	9
Diarrhea	23	8
Vomiting	12	4
Anosmia	11	4
